# Functional Deficits and Structural Changes Associated With the Visual Attention Network During Resting State in Adult Strabismic and Anisometropic Amblyopes

**DOI:** 10.3389/fnhum.2022.862703

**Published:** 2022-05-18

**Authors:** Hao Wang, Minglong Liang, Sheila G. Crewther, Zhengqin Yin, Jian Wang, David P. Crewther, Tao Yu

**Affiliations:** ^1^Southwest Hospital/Southwest Eye Hospital, Third Military Medical University, Army Military Medical University, Chongqing, China; ^2^Key Lab of Visual Damage and Regeneration and Restoration of Chongqing, Chongqing, China; ^3^Department of Radiology, Southwest Hospital, Army Medical University, Chongqing, China; ^4^Department of Radiology, Aviation Medical Evaluation and Training Center of Airforce in Hangzhou, Hangzhou, China; ^5^School of Psychology and Public Health, La Trobe University, Melbourne, VIC, Australia; ^6^Centre for Human Psychopharmacology, Swinburne University of Technology, Melbourne, VIC, Australia

**Keywords:** strabismic amblyopia, anisometropic amblyopia, visual attention network, resting-state functional MRI, structural MRI

## Abstract

Our previous study has shown impaired blood oxygen level-dependent (BOLD)/functional magnetic resonance imaging (fMRI) activation of the visual attention network in strabismic amblyopia (SA). However, there has been no comparison of resting state fMRI activation and functional connectivity (FC) in brain regions of interest (ROIs) along the visual attention network including visual cortex (V1), intraparietal sulcus (IPS), and frontal eye fields (FEFs) during closed eye resting across the SA (*n* = 20, 13LE), or anisometropic amblyopes (AA) (*n* = 20, 13LE) groups. Hence, we compared, gray matter volume (GMV), amplitude of low frequency fluctuations (ALFFs), regional homogeneity (ReHo), and FC in the left and right hemisphere ROIs of the visual attention network in SA, AA, and healthy controls (HCs) (*n* = 21). Correlation analyses of corrected visual acuity (cVA) of amblyopic eye and MRI results were also performed and showed that the LogMAR cVA of the amblyopic eye positively correlated with right zALFF and zReHo FEF of SA and right IPS of AA only. GMV of both left and right hemisphere V1 areas was significantly greater but ALFF was significantly lower for SA compared to AA and HC groups. zALFF and zReHo analyses in the AA and SA groups indicated significantly higher activation than that in the HC group in the right FEF and IPS but lower than that in the HC group in the left FEF, and only the SA group showed lower activation in both V1 areas than the HC group. FC values of the right FEF–left V1, right FEF–right V1, and right FEF–right IPS pathways in the SA and AA groups were also significantly higher than those in the HC group whereas all other FC values were non-significant. Thus, this study indicates that even during resting-state the visual attention network function is impaired in SA and AA participants with only right hemisphere FEF showing significant activation in SA and IPS in AA suggesting that the slower saccade activation times characteristic of amblyopic eyes lead to the dominant eye controlling activation of the visual attention network.

## Introduction

Amblyopia is traditionally defined as a congenital anomaly in the spatial–temporal processing of visual information (Noorden, 1977; Ciuffreda et al., 1978b; Hamasaki and Flynn, 1981; Asper et al., 2000a,b) occasioned by abnormal visual experience early in life associated with strabismus (misalignment of the visual axes of the two eyes), anisometropia (refractive differences between the eye), and/or visual deprivation (Webber and Wood, 2005). Studies such as those of noted that the saccadic reaction times to visual stimuli of strabismic amblyopic eyes were significantly longer than those by the dominant eye (Ciuffreda et al., 1978a; Hamasaki and Flynn, 1981). Strabismic amblyopia (SA) eyes were also known to show eye movement problems such as unstable fixation, and pursuit difficulty (Asper et al., 2000a; Kanonidou et al., 2014). More recently, studies have described similar temporal processing and eye movement deficits associated with anisometropic amblyopia (Niechwiej-Szwedo et al., 2010, 2011a,b). Thus, given that eye movements and attention are intrinsically linked (Wurtz et al., 1982), and visual attention to a particular stimulus or location is known to selectively amplify the activity of sensory neurons (Goldberg and Wurtz, 1972), it is not surprising that visual attention and perception are often anomalous through either strabismic and anisometropic amblyopic eyes making treatment for amblyopia difficult to achieve in older children and adults. Indeed a systematic review and meta-analysis of recent research into dichoptic training, perceptual learning and video gaming has noted that “some participants may benefit from the new treatments and aid some recovery of visual acuity through the amblyopic eye but not binocular stereo acuity” (Murphy et al., 2015; Tsirlin et al., 2015), suggesting that aspects of dynamic attentional function through the amblyopic eye continue to be defective.

Visual attention deficits have been reported in many previous studies of amblyopia (Asper et al., 2000a,b; Simmers et al., 2003; Ho et al., 2006; Popple and Levi, 2008), with evidence of temporal processing inadequacies and inability of the amblyopic eye to initiate eye movements and shift attention as rapidly as the dominant non-amblyopic eye (Goldberg and Wurtz, 1972; Noorden, 1977). Sharma et al. (2000) also reported that SA patients undercounted features presented to the amblyopic eye, although the attentional cueing effects were the same as for controls while Thiel and Sireteanu (2009) noted line bisection task deficits in SA patients similar to that seen in neuropsychological cases of hemineglect. Tsirlin et al. (2018) also utilized a conjunction visual search task that required top-down attentional processes to provide evidence of functional deficits in visual attention in amblyopes (Tsirlin et al., 2018). However, details of aspects of the neural mechanisms underlying visual attention deficits in amblyopia are still largely unknown.

Perceptual learning treatment for visual attention has been reported to be effective in treating amblyopia by improving visual acuity or restoring stereoscopic function monocularly for a short time (Huang et al., 2014; Tsirlin et al., 2015). However, when training is discontinued and binocular vision is available, the slower activation of voluntarily directed eye movements (and hence attention) through the strabismic or anisometropic amblyopic eye is likely to favor the dominant, faster to activate eye and reinduce amblyopia as an adaptive measure to prevent double vision, and temporal mismatch of images (Harrad et al., 1996), and potentially most importantly splitting of ocularly driven attention.

Such a potential scenario is supported by our previous brain imaging study that demonstrated that activation of the visual attention network driven by the strabismic amblyopic eye in performance on goal-directed and stimulus-driven visual attention tasks is reduced compared to that of the non-amblyopic eye (Wang et al., 2015, 2017). However, functional connectivity (FC) of the visual attention network in strabismic amblyopic patients in the resting state has not been addressed to date suggesting the need for investigation of FC of the cortical network structure in visual attention-related areas of strabismic amblyopes. On the other hand, there are a number of reports of abnormal connections in extra striate visual spatial network cortices and between hemispheres differences in anisometropic amblyopia during resting state functional magnetic resonance imaging (fMRI) (Ding et al., 2013; Joly and Franko, 2014; Wang et al., 2014).

Visual attention is primarily driven by the dorsal cortical visual stream, which includes the primary visual cortex (V1), middle temporal area (MT), intraparietal sulcus (IPS), and frontal eye field (FEF) (Gilbert and Li, 2013; Liu et al., 2014; Patel et al., 2015) and frontal cortical areas with the three longitudinal fasciculi filling a significantly larger volume in the right hemisphere (Corbetta and Shulman, 2002). V1 is the initial area in the cortical visual processing system, and feeds to MT, although MT in developing primate also receives other subcortical fast visual-related signals *via* Pulvinar with projection of signals to IPS and FEF (Mundinano et al., 2019; Kwan et al., 2021). IPS is known to be critically involved in contralateral spatial selective attention (Gillebert et al., 2011) and FEF contribute to fixation, saccade, pursuit, and vergence movements and cognitive processes such as attentional orienting, visual awareness, and perceptual modulation (Vernet et al., 2014; Matsubayashi et al., 2018).

Thus, in the present study, we chose to evaluate groups of strabismic amblyopic (SA) and anisometropic amblyopic (AA) patients and compared them with healthy controls (HCs). Resting-state functional magnetic resonance imaging (rs-fMRI) and high-resolution structural MRI scans were performed with each subject. The blood oxygen level-dependent (BOLD) and structure-related signals in visual attention-related regions of interest (ROIs), including the gray matter volume (GMV), amplitude of low frequency fluctuations (ALFFs), regional homogeneity (ReHo), and FC among the six ROIs. We expected to find functional and structural deficits in the ipsilateral visual attention network in the two amblyopic groups in the resting state given that eye movements and shifts in attention are predominantly driven by the ipsilateral hemisphere (Troost et al., 1972). Such information should progress understanding of visual attention network deficits in those with amblyopia and highlight the hemispheric limitations on neural control of initiation of eye movements and shifts in attention.

## Materials and Methods

### Participants

This study adhered to the Declaration of Helsinki principles. Amblyopia patients were recruited from the eye department of Southwest Hospital, and all subjects gave their written informed consent. The criteria were in accordance with the expert consensus on amblyopia diagnosis (Rubin and Nelson, 1993). All subjects received detailed ophthalmological examinations, including best corrected visual acuity (cVA), refraction, slit lamp examination, ophthalmoscopy, optical coherence tomography, and ocular motility, to ensure suitability for this study.

The inclusion criteria included male or female patients aged 16–40 years old with strabismic or anisometropic amblyopia. Subjects with SA had to have undergone strabismus surgery at least 3 years ago, have no history of neurological or psychiatric disorders, be right-handed and have no history of other ocular diseases. Subjects were screened for MRI contraindications to ensure the safety of the fMRI examination. The clinical details of the SA and AA subjects are shown in Table 1.

**TABLE 1:** Clinical details of the strabismic and anisometropic amblyopic subjects.

SU	SX	AG	AE	LM/L	LM/R	Refraction (L)	Refraction (R)
SA1	F	22	L	1.00	0.00	+0.50 DS/−1.00 DC × 5°	−0.25 DS
SA2	M	29	R	−0.08	0.52	−0.50 DC × 35°	−0.50 DC × 150°
SA3	F	17	L	1.30	0.00	−0.50 DS/−0.50 DC × 55°	−1.75 DS/−0.50 DC × 105°
SA4	F	26	L	0.40	0.00	+0.75 DS	+1.00 DS/−1.25 DC × 165°
SA5	M	19	R	−0.08	2.00	+0.50 DS	+0.50 DS/−1.5 DS × 5°
SA6	F	16	L	0.82	0.00	+2.00 DS/−1.25 DC × 125°	+1.00 DS
SA7	M	31	R	0.00	0.70	−0.75 DS/−0.50 DC × 90°	−0.75 DS
SA8	F	25	L	0.30	0.00	+2.75 DS/−1.75 DC × 165°	+2.75 DS
SA9	M	21	L	1.30	−0.08	+1.00 DS/−0.75 DC × 25°	+0.25 DS/−0.25 DC × 180°
SA10	F	18	R	0.00	1.00	−0.75 DS	+1.50 DS
SA11	M	28	R	0.00	1.00	0	+0.25 DS/−0.75 DC × 125°
SA12	F	32	R	0.00	1.22	−0.50 DS/−0.75 DC × 90°	+1.00 DS/−0.25 DC × 175°
SA13	F	25	L	1.05	0.05	+1.00 DS/−1.50 DC × 5°	−0.25 DS/−0.50 DC × 155°
SA14	M	25	L	1.22	0.00	+1.50 DS/−1.00 DC × 15°	+0.75 DS/−0.50 DC × 160°
SA15	M	22	R	−0.08	1.00	+0.50 DS	+1.25 DS
SA16	F	19	L	0.52	−0.08	+1.25 DS/−1.75 DC × 165°	+1.00 DS/−0.25 DC × 40°
SA17	M	16	L	2.00	−0.08	0	+1.00 DS/−1.25 DC × 85°
SA18	F	17	L	1.00	0.00	+1.75 DS/−0.50 DC × 5°	+1.25 DS/−0.50 DC × 155°
SA19	M	27	L	1.30	−0.08	+1.75 DS/−1.00 DC × 80°	+1.00 DS
SA20	M	16	L	1.30	0.00	−2.00 DS/−0.75 DC × 180°	+1.00 DS
AA1	F	29	R	0.00	0.52	−2.00 DS/−0.75 DC × 180°	+3.00 DS
AA2	F	26	L	0.52	0.00	+4.25 DS/−1.25 DC × 167°	−3.50 DS/−1.00 DC × 45°
AA3	M	17	L	0.30	−0.08	+3.25 DS/−2.00 DC × 175°	0
AA4	F	22	R	0.00	1.10	0	+4.00 DS/−0.50 DC × 170°
AA5	M	17	L	0.82	−0.08	+8.00 DS/−2.00 DC × 15°	+0.75 DS/−0.75 DC × 165°
AA6	F	17	L	0.70	0.05	+3.50 DS	−1.25 DS
AA7	M	23	L	0.30	0.00	+3.50 DS/−1.25 DC × 180°	−1.25 DS
AA8	M	25	R	0.00	1.00	0	+3.25 DS/−0.50 DC × 170°
AA9	F	17	L	0.70	−0.08	+3.75 DS/−1.25 DC × 5°	0
AA10	F	22	R	−0.08	1.30	+0.75 DS	+3.75 DS/−0.50 DC × 5°
AA11	M	21	L	0.82	0.10	+7.50 DS/−1.50 DC × 20°	+1.75 DS/−1.50 DC × 165°
AA12	M	19	L	0.52	0.05	+4.75 DS/−1.75 DC × 180°	−2.50 DS
AA13	F	23	L	0.52	0.00	+3.75 DS/−1.25 DC × 180°	−2.25 DS/−0.50 DC × 5°
AA14	F	23	L	1.00	0.05	+2.50 DS/−1.50 DC × 170°	−0.25 DS
AA15	M	19	R	−0.18	0.82	0	+6.50 DS/−0.75 DC × 165°
AA16	F	25	R	0.00	0.70	−0.25 DS	+6.00 DS/−0.75 DC × 125°
AA17	M	16	L	0.82	−0.08	+5.50 DS/−1.00 DC × 25°	+1.25 DS/−0.50 DC × 5°
AA18	F	19	R	0.00	0.82	+1.75 DS/−1.00 DC × 10°	+4.00 DS/−1.50 DC × 180°
AA19	F	22	L	0.40	0.05	+3.50 DS/−2.25 DC × 10°	+0.50 DS/−0.75 DC × 170°
AA20	F	21	L	0.82	0.00	+5.50 DS/−1.75 DC × 175°	+0.50 DS

*SU, subject; SX, sex; AG, age; AE, amblyopic eye; LM/L, LogMAR in the left eye; LM/R, LogMAR in the right eye.*

### MRI Data Acquisition

All MR images were acquired at the Department of Radiology of Southwest Hospital *via* a 3.0 Tesla MR scanner (Trio Tim system, Siemens, Germany) equipped with an eight-channel phase array head coil. Foam padding was used to limit head movement and reduce scanner noise. T1-weighted structural images were acquired using a magnetization-prepared rapid gradient echo imaging sequence with the following scan parameters: repetition time (TR) = 2530 ms, echo time (TE) = 2.34 ms, flip angle (FA) = 7°, matrix = 192 × 256, field of view (FOV) = 256 × 256 mm^2^, slice thickness = 1 mm, slice gap = 0.5 mm, and 192 sagittal slices. The total scan time was approximately 20 min. rs-fMRI data were acquired using an echo-planar imaging (EPI) sequence with the following parameters: TR = 2000 ms, TE = 30 ms, FA = 90°, FOV = 192 × 192 mm^2^, matrix = 64 × 64, slice thickness = 3 mm, no slice gap, 36 axial slices, and 240 volumes. All subjects were required to keep their eyes closed and their heads stable and to remain awake during the scanning.

### Structural MRI Processing

The structural MRI data from the T1-weighted images were processed using the Voxel-based Morphometry 8 toolbox (VBM8^1^) based on Statistical Parametric Mapping (SPM12). Each subject’s structural image set was spatially normalized to the Montreal Neurological Institute (MNI) space by a high-dimensional Diffeomorphic Anatomical Registration Through Exponentiated Lie Algebra (DARTEL) algorithm. The spatially normalized structural images were then segmented into gray matter (GM), white matter (WM), and cerebrospinal fluid (CSF), and probability plots of GM and WM were generated. Then, the probability plot of GM was smoothed with an 8-mm full-width at half-maximum (FWHM) isotropic Gaussian kernel, and the smoothed GM images were resampled to a 3 mm × 3 mm × 3 mm voxel size. Finally, the ROI masks of the V1, IPS, and FEF were used to extract the average GMV in the ROIs by REST plus.

### Resting-State Functional Magnetic Resonance Imaging Data Processing

#### Functional Data Pre-processing

The rs-fMRI data were processed using the Data Processing Assistant for Resting-State fMRI toolkit (DPARSF^2^) based on SPM12.^3^ The rs-fMRI data were pre-processed using the following procedures. The first 10 volumes were discarded to remove the initial transient signal fluctuations. Then, slice timing and realignment for head motion correction were performed for the remaining volumes. Any participant with a head motion more than 1.0 mm translation or 1.0° rotation on any axis was excluded. The eligible realigned data were then spatially normalized in the MNI space using the average template made by the DARTEL approach with their own T1-weighted images. The global mean signal, WM signal, and CSF signal were removed from the normalized data by multiple linear regression. Subsequently, the regressed images were smoothed with a Gaussian kernel of 6 mm at FWHM. Finally, linear detrending and temporal bandpass filtering (0.01–0.08 Hz) were applied to reduce the effect of low-frequency drifts and high-frequency physiological noise.

#### Amplitude of Low-Frequency Fluctuations, Regional Homogeneity, and Functional Connectivity Processing of Region of Interest Data

We defined 3 × 2 ROIs related to dorsal visual attention networks, namely, the V1, IPS, and FEF in the two hemispheres, by anatomical location based on standard MRI structural coordinates in MNI space. For both V1 ROIs, which were defined as Brodmann area 17 *via* an anatomical MRI (SPM toolbox Xjview) mask (Brodmann area 17 is widely equated to the V1). The IPS ROI was defined as the sulcus between the superior parietal lobule and inferior parietal lobule, with the anterior boundary being the postcentral sulcus and the posterior boundary being the transverse occipital sulcus. We manually built the IPS mask based on these anatomical coordinates and by selecting the GM within this area to be the ROI. The FEF is located around the junction of the precentral gyrus and the middle frontal gyrus and was also localized for our purposes based on MNI coordinates from previous human fMRI research. An 8 mm × 8 mm × 8 mm area in each hemisphere was specified to be the mask for the FEF ROI. These coordinates are similar to those used by Secen et al. (2011).

Amplitude of low frequency fluctuation and ReHo values were calculated by the Resting-State fMRI Data Analysis Toolkit plus (REST plus^4^) with the Kendall concordance algorithm. The ROI masks of the V1, IPS, and FEF were used to extract the average ALFF and ReHo values in the ROIs by REST plus. The FC values among the six ROIs were also calculated by REST plus. All ALFF, ReHo, and FC values were subjected to Fisher *r*-to-*z* transformation to improve normality. Peak-level topological false discovery rate (FDR) correction was used to correct for multiple comparisons. The BrainNet Viewer^5^ toolbox was selected to show the statistical maps of the cortex.

### Statistical Analysis

All statistical analyses were performed using SPSS software (version 25.0; SPSS, Inc., Chicago, IL, United States) with statistical significance set at 0.05. One-way ANOVA was used to analyze VBM values and Fisher *r*-to-*z* transformed values of the ALFF, ReHo, and FC among the three groups. All ANOVAs underwent Bonferroni correction to control type 1 error. *Post hoc* testing was performed using the LSD method in SPSS, and statistical significance was set at *P* < 0.05. Bonferroni correction was used to control for multiple comparisons, and the adjusted *P*-value was set at *P_adj* < 0.05 in the preceding ANOVA. Group differences in age and years of education in the three groups were compared using ANOVA. Sex and group differences in laterality of amblyopic/non-dominant eye differences were analyzed with Chi-square tests. For cVA of the amblyopic/fellow/non-dominant/dominant eye, Wilcoxon’s signed rank tests and Mann–Whitney *U* tests were performed to compare the paired and independent data, respectively. Spearman’s rank correlation analysis was used to explore the correlation between the ROIs MRI results and the LogMAR cVA values of amblyopic eyes.

## Results

### Demographic and Clinical Variables

The demographic characteristics and clinical variables are presented in Table 2. No significant differences were found in age, sex, education, or laterality of the amblyopic/non-dominant eye among the SA, AA, and HC groups. In the AA and SA groups, the cVA of the amblyopic eye was significantly lower than that of the fellow eye, whereas there was no significant difference in cVA between the dominant and non-dominant eyes in the HC group. In addition, the cVA of the amblyopic eye was significantly lower than that of the non-dominant eye of the HCs.

**TABLE 2:** Demographic and clinical variables of the participants.

Characteristics	SA (*n* = 20)	AA (*n* = 20)	HC (*n* = 21)	*P*-value
Age (years)	22.55 ± 5.10	21.15 ± 3.44	21.86 ± 2.87	0.546
Sex (male/female)	10/10	8/12	12/9	0.545
Education (years)	12.59 ± 2.38	11.95 ± 1.94	12.25 ± 2.15	0.630
Amblyopia/non-dominant eye of control (left/right)	13/7	13/7	12/9	0.834
cVA (LogMAR)				
Amblyopic eye (non-dominant eye of control)	1.05 ± 0.44	0.73 ± 0.26	−0.02 ± 0.02	<0.001
Fellow eye (dominant eye of control)	−0.03 ± 0.04	−0.01 ± 0.06	−0.02 ± 0.02	0.828
*P*-value	<0.001	<0.001	0.985	–

### Changes in Gray Matter Volume in Visual Attention-Related Cortical Areas

**FIGURE 1:**
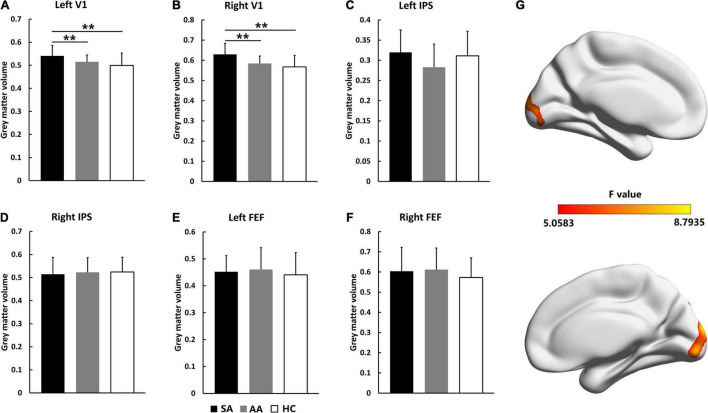
Gray matter volume comparisons across the SA, AA, and HC groups in the left and right FEF, IPS, and V1 areas. **(A)** GMV comparison in the left V1; **(B)** GMV comparison in the right V1; **(C)** GMV comparison in the left IPS; **(D)** GMV comparison in the right IPS; **(E)** GMV comparison in the left FEF; **(F)** GMV comparison in the right FEF. **(G)** Statistical differences (*F*-values) in VBM values across brain areas in the three groups (**P_adj < 0.01).

The GMV changes in visual attention-related cortical areas were analyzed by VBM values. As shown in Figure 1, VBM values were compared in the left and right V1, IPS, and FEF areas. For the AA, SA, and HC groups, the significantly different brain areas based on VBM are shown in Figure 1G (*F* = 5.0583–8.7935, *P_adj* < 0.05, FDR correction). VBM ANOVA was also performed with Bonferroni correction in the left V1 (*F* = 6.381, *P_adj* = 0.007) and right V1 (*F* = 7.659, *P_adj* = 0.001). In both V1 areas, the VBM values in the SA group were significantly higher than those in the AA and HC groups, but the VBM values in the AA group were not significantly different from those in the HC group. These results indicated that the GMV in the SA group in the V1 increased and that the GMV in the AA group in the V1 was normal (Figures 1A,B). Both the FEF and IPS areas were not significantly different across the three groups, and therefore, the GMV values in the SA and AA groups were normal (Figures 1C–F).

### Resting-State Activation Intensity in Visual Attention-Related Cortical Areas

**FIGURE 2:**
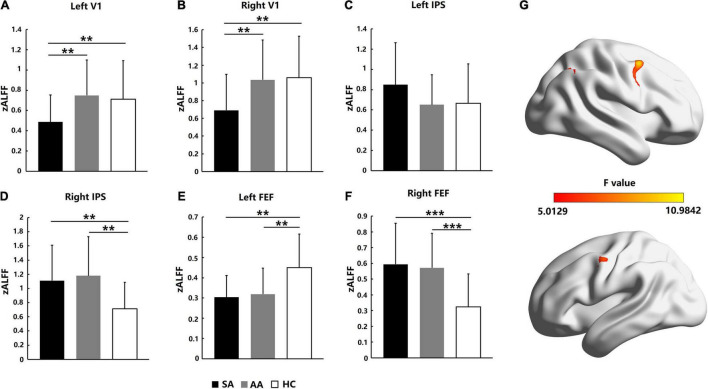
Resting-state activation intensity comparison across the SA, AA, and HC groups in the left and right FEF, IPS, and V1 areas. **(A)** zALFF comparison in the left V1; **(B)** zALFF comparison in the right V1; **(C)** zALFF comparison in the left IPS; **(D)** zALFF comparison in the right IPS; **(E)** zALFF comparison in the left FEF; **(F)** zALFF comparison in the right FEF. **(G)** Statistical differences (*F*-values) in ALFF across brain areas in the three groups (**P_adj < 0.01, ***P_adj < 0.001).

Resting-state activation intensities in visual attention-related cortical areas were analyzed by ALFF values. As shown in Figure 2, for the AA, SA, and HC groups, the significantly different brain areas based on ALFF values are shown in Figure 2G (*F* = 5.0129–10.9842, *P_adj* < 0.05, FDR correction). The Fisher-transformed ALFF values (zALFF) were compared across ROIs. Differences in zALFF values with a Bonferroni correction were identified in the left V1 (*F* = 5.298, *P_adj* = 0.036) and right V1 (*F* = 5.304, *P_adj* = 0.044). Resting-state activation intensities in the SA group were significantly lower than those in the AA and HC groups (Figures 2A,B).

ANOVA with zALFF values was also performed with a Bonferroni correction in the left IPS area (*F* = 1.494, *P_adj* = 1) and right IPS area (*F* = 5.304, *P_adj* = 0.039). No significant differences in zALFF values were found in the left IPS area of the parietal lobe (Figure 2C), but the resting-state activation intensities in both the SA and AA groups were significantly higher than those in the HC group in the right IPS (Figure 2D).

In the left FEF area of the frontal lobe, ANOVA was performed with a Bonferroni correction with the zALFF values (*F* = 7.088, *P_adj* = 0.008). The zALFF values in both the SA and AA groups were significantly lower than those in the HC group, which indicated that the resting-state activation intensities in the left FEF in both the SA and AA groups decreased (Figure 2E). In the right FEF area, ANOVA with the zALFF values was performed with a Bonferroni correction (*F* = 9.151, *P_adj* < 0.001). The zALFF values in both the SA and AA groups were significantly higher than those in the HC group, which indicated that the resting-state activation intensities in the right FEF in both the SA and AA groups increased (Figure 2F).

### Homogeneity in Regional Activation in Visual Attention-Related Cortical Areas

**FIGURE 3:**
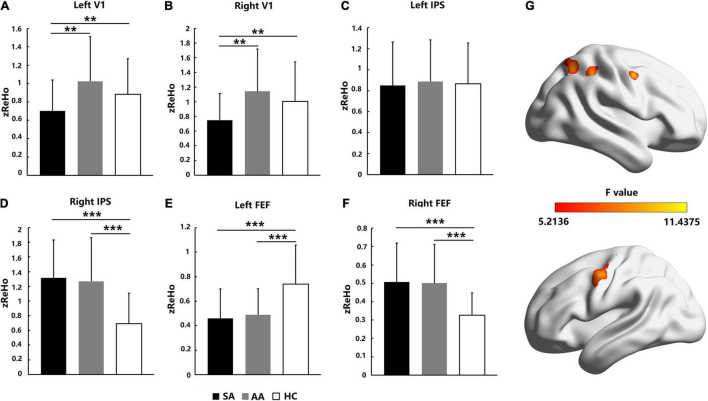
Homogeneity in regional activation comparison across the SA, AA, and HC groups in the left and right FEF, IPS, and V1 areas. **(A)** zReHo comparison in the left V1; **(B)** zReHo comparison in the right V1; **(C)** zReHo comparison in the left IPS; **(D)** zReHo comparison in the right IPS; **(E)** zReHo comparison in the left FEF; **(F)** zReHo comparison in the right FEF. **(G)** Statistical differences (*F*-values) in ReHo across brain areas in the three groups (**P_adj < 0.01 and ***P_adj < 0.001).

Homogeneity in regional activation in visual attention-related cortical areas was analyzed with ReHo values. As shown in Figure 3, for the AA, SA, and HC groups, the significantly different brain areas based on ReHo are shown in Figure 3G (*F* = 5.2136–11.4375, *P_adj* < 0.05, FDR correction). The Fisher-transformed ReHo values (zReHo) were compared in the left and right V1, IPS, and FEF areas. ANOVA with the zReHo values was also performed with a Bonferroni correction in the left V1 (*F* = 6.603, *P_adj* = 0.036) and right V1 (*F* = 5.608, *P_adj* = 0.042). The zReHo values in the SA group in both V1 areas were significantly lower than those in the AA and HC groups, which indicated that the homogeneity in regional activation in both V1 areas in the SA patients decreased. However, the homogeneity in regional activation in both V1 areas in the AA patients was normal (Figures 3A,B).

In the IPS area of the parietal lobe, ANOVA was performed with a Bonferroni correction in the left IPS (*F* = 0.068, *P_adj* = 1) and right IPS (*F* = 9.338, *P_adj* < 0.001). No significant differences in zReHo values were found in the left IPS, which indicated that homogeneity in regional activation in the left IPS in both SA and AA patients was normal (Figure 3C). However, both the SA and AA zReHo values in the right IPS were significantly higher than those in the HC group, which indicated that homogeneity in regional activation in the right IPS in both the SA and AA patients increased (Figure 3D).

In the FEF areas of the frontal lobe, ANOVA was performed with a Bonferroni correction in the left FEF (*F* = 9.151, *P_adj* < 0.001) and right FEF (*F* = 10.330, *P_adj* < 0.001). In the left FEF, the zReHo values in both the SA and AA groups were significantly lower than those in the HC group, which indicated that homogeneity in regional activation in the left FEF in both the SA and AA groups decreased (Figure 3E). In the right FEF area, the zReHo values in both the SA and AA groups were significantly higher than those in the HC group, which indicated that homogeneity in regional activation in the right FEF in both the SA and AA groups increased (Figure 3F).

### Functional Connectivity Among Visual Attention-Related Cortical Areas

The FC among the FEF, IPS, and V1 areas was compared in the SA, AA, and HC groups. ANOVA was performed with a Bonferroni correction for all FC measures. No significant differences were found in most FC measures, with the exceptions of right FEF–right IPS (*F* = 6.256, *P_adj* = 0.042), right FEF–left V1 (*F* = 6.614, *P_adj* = 0.039), and right FEF–right V1 (*F* = 6.897, *P_adj* = 0.033; see Table 3).

**TABLE 3:** Summary of ANOVA *F*-values and *P_adj* values for functional connectivity measures.

	Left V1	Right V1	Left IPS	Right IPS	Left FEF	Right FEF
Left V1	/					
Right V1	*F* = 0.244 *P_adj* = 1	/				
Left IPS	*F* = 2.565 *P_adj* = 1	*F* = 2.918 *P_adj* = 1	/			
Right IPS	*F* = 0.390 *P_adj* = 1	*F* = 0.520 *P_adj* = 1	*F* = 0.143 *P_adj* = 1	/		
Left FEF	*F* = 4.703 *P_adj* = 0.195	*F* = 5.358 *P_adj* = 0.105	*F* = 0.079 *P_adj* = 1	*F* = 0.552 *P_adj* = 1	/	
Right FEF	*F* = 6.614 *P_adj* = 0.039	*F* = 6.897 *P_adj* = 0.033	*F* = 2.981 *P_adj* = 0.953	*F* = 6.256 *P_adj* = 0.042	*F* = 0.483 *P_adj* = 1	/

**FIGURE 4:**
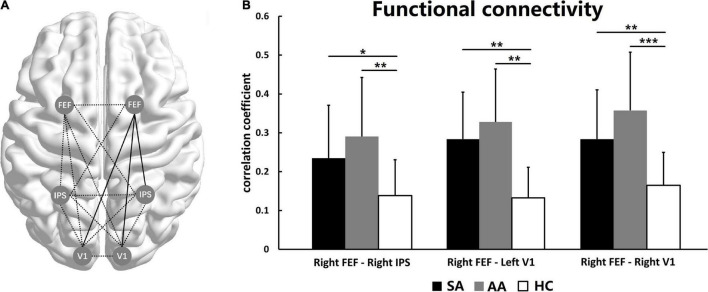
Functional connectivity among the left and right FEF, IPS, and V1. **(A)** The functional network among the ROIs. The full lines represent FC with a significant difference, and the dotted lines represent no significant difference. **(B)** FC comparisons in three pathways show significant differences (*P_adj < 0.05, **P_adj < 0.01, ***P_adj < 0.001).

As shown in Figure 4, the right FEF was a core ROI in the visual attention network. The FC between the right FEF and three other ROIs was significantly changed, including the FC with the right IPS, left V1, and right V1. Therefore, the FC between the right FEF and the right parietal lobe and both occipital lobes significantly changed (Figure 4A). The statistics showed that the FC values in both the SA and AA groups were significantly higher than those in the HC group in all three pathways. These results indicated that FC in the three pathways significantly increased in the SA and AA groups (Figure 4B).

### Correlation Analysis of Regions of Interest MRI Results and Corrected Visual Acuity Value of Amblyopic Eye

**FIGURE 5:**
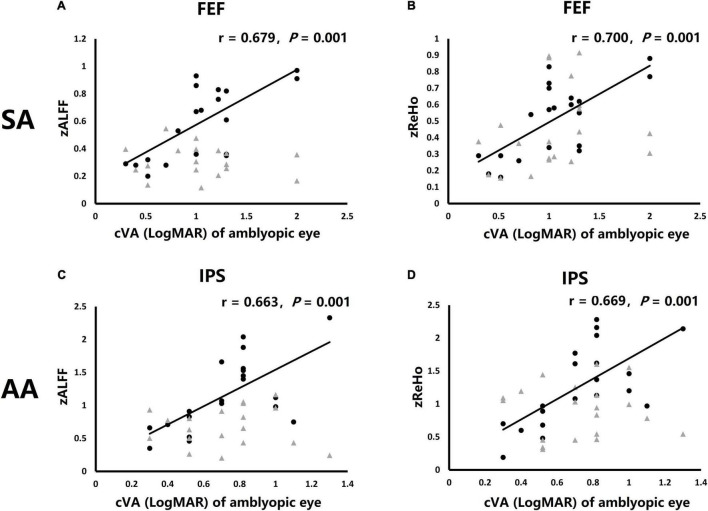
Correlations of clinical visual acuity (cVA) value of amblyopic eye were significant correlations with fMRI activation in particular ROIs. **(A)** Correlation of cVA of SA group and zALFF of both FEF. **(B)** Correlation of cVA of SA group and zReHo of both FEF. **(C)** Correlation of cVA of AA group and zALFF of both IPS. **(D)** Correlation of cVA of AA group and zReHo of both IPS. Black dots: right hemisphere ROI, gray triangles: left hemisphere ROI (no significantly correlation).

To explore the relationship of clinical measures and ROIs MRI results, Spearman’s rank correlation analysis was used to analyze the correlation coefficient of amblyopic eye cVA and ROIs MRI results including GMV, zALFF, and zReHo. The amblyopic eye cVA showed significant positive correlations with the zALFF and zReHo value of right FEF in SA group (*r* = 0.679, *P* = 0.001, Figure 5A; *r* = 0.700, *P* = 0.001, Figure 5B) while the amblyopic eye cVA of the AA group, showed significant positive correlations with the zALFF and zReHo value of right IPS (*r* = 0.679, *P* = 0.001, Figure 5C; *r* = 0.700, *P* = 0.001, Figure 5D) indicating that poor acuity in the amblyopic eyes correlated with high activation in only one area per amblyopic group.

## Discussion

This study has evaluated BOLD activation during resting state conditions in the visual attention network ROI including V1, IPS, and FEF in groups with SA and AA and HC demonstrating that activation intensities and resting FC among are deficient in both SA and AA participants compared to HC. We also investigated the structural changes in visual attention-related brain areas and found that the GMV in the V1 was anomalous in SA patients but not in AA patients. Furthermore, zALFF and zReHo measures for the right FEF of SA and right IPS of AA, showed positive correlation with LogMAR cVA of amblyopic eye indicating that poor acuity in the amblyopic eyes correlated with high activation in one area only in each subtype of amblyopia.

In our earlier studies we have shown that the function of both the FEF and IPS areas in SA patients is markedly decreased for goal-driven and stimulus-driven attention tasks, compared with that in HCs (Wang et al., 2017). Under resting-state conditions, we have now found that the left FEF areas that are involved in driving eye movements and hence visual attention to salient stimuli in both SA and AA patients, also showed lower activation intensity and homogeneity than those in HCs, but that the right FEF and IPS showed higher activation intensity and homogeneity than those in HCs presumably because all the amblyopic subjects had experienced abnormal visual experience through the amblyopic eye since infancy and the right brain areas associated with the visual attention network is volumetrically and functionally greater in the right cerebral hemispheres, and is more dominant than that in the left hemisphere (Heilman et al., 1983; Shulman et al., 2010; Thiebaut de Schotten et al., 2011). Therefore, high levels of activation in the FEF and IPS in the right hemisphere could be due to compensatory increases in resting-state situations that enhance the visual attention function of amblyopia patients. The decreased activation intensity and homogeneity in the left FEF was also possibly due to suppression of the attention network by the dominant right cerebral hemisphere and the fact that both the SA and AA groups included 13/20 left eye amblyopes and it is known that eye movements and shifts in attention are predominantly driven by the ipsilateral hemisphere (Troost et al., 1972).

Currently there are a number of studies suggesting that the functional activation in the V1 are abnormal in patients with amblyopia (Jia et al., 2010; Li et al., 2013; Restani and Caleo, 2016), with visual attention modulation and goal-driven attention tasks in SA patients is reduced in V1 (Hou et al., 2016; Wang et al., 2017). There are also suggestions that V1 areas and the corpus callosum connecting them does not mature normally under abnormal visual conditions such as amblyopia (Restani and Caleo, 2016). Similarly in this resting-state study, functional activation in both V1 areas was also defective in SA patients while our VBM results suggests that the GMV in the V1 was increased in SA patients but not AA patients. The AA group results accorded with earlier AA research (Liang et al., 2019), and a higher GMV in SA group often represents unmatured development of the cerebral cortex (Barlow, 1975; Giedd et al., 1999; Sowell et al., 2004). Furthermore, this finding could just reflect that V1 area in SA patients is even more monocular with less binocular connections than in AA and HC patients and hence did not show normal decrease in synapse number early in life (Zhang et al., 2005; Jenks and Shepherd, 2020).

Functional connectivity values among visual attention areas were dramatically perturbed in both SA and AA patients compared with health controls. Thompson et al. (2019) have speculated that the higher-level processing areas of the brain including the striate and extra striate areas are involved in the neural suppression of amblyopia in the visual cortex. We propose that visual attention network deficits associated with eye movement and saccade initiation are the key reason for functional suppression in the visual cortex. In this study, the increased connectivity of the SA and AA groups between the left/right V1 areas and the right FEF may be a monocular indirect compensatory effect for the difference in the temporal activation of eye movements and hence of involuntary bottom-up attention in the ROI in the visual attention network. FC between the right FEF and right IPS was also dramatically increased in both groups of amblyopic patients remembering that the right visual attention network is stronger than that in the left hemisphere (Shulman et al., 2010; Posner, 2012), especially with regard to salience information. Thus, it is possible that the increased connectivity between the right FEF and right IPS is due to the usual suppression of the amblyopic eyes (13 left, 7 right in both groups in this study) within the visual attention network.

In the zALFF and zReHo comparisons, the SA and AA groups showed a high degree of similarity in both the FEF and IPS areas. This finding indicates that the impairment of the visual attention network was similar in the two kinds of amblyopia. However, both V1 areas were exceptions in both groups. As the primary visual cortex, the V1 area receives visual input from both eyes and is the original site of breakdown of spatial frequency processing and binocular function in amblyopia (Crewther and Crewther, 1990; Kiorpes and McKee, 1999; Kiorpes et al., 2003). The different manifestations in V1 in resting condition is likely to be due to a lifetime of abnormal visual experience with the ocular misalignment of a strabismus generating unmatched spatial and temporal visual input from the amblyopic eye, and with the different magnifications in anisometropia generating unclear visual input of larger magnifications from the more amblyopic eye (Levartovsky et al., 1998). A previous study also found that SA resulted in more serious visual deficits and greater suppression from the non-amblyopic eyes than in anisometropic amblyopia (Agrawal et al., 2006; Jia et al., 2010) and SA is usually characterized by greater oculomotor abnormalities than AA. Therefore, it is likely that the SA group showed greater functional and structural anomalies, than did the AA group.

The FEF area and visual attention network also play a key role in eye movement control (Wang et al., 2015; Mahon et al., 2018) so at least for the SA eyes that have long been reported to show eye movement problems such as unstable fixation, saccade delay, and pursuit difficulty (Helveston and Von Noorden, 1967; Asper et al., 2000a; Kanonidou et al., 2014). It is likely that strabismus is associated with considerable eye movement deficits in the development stage (Schor, 1975). This would be expected to more directly affect the development of V1 areas compared with other ROIs because of the close relationship to visual signal processing. This may explain why we found that the GMV of V1 was greater in the SA group than in the AA and HC groups and may also explain the greater eye movement deficits of SA eyes in adult patients.

In correlation analysis, clinical visual acuity of strabismic amblyopic eye, measured as LogMAR cVA showed positive correlation with activation of the right FEF in SA possibly because the right FEF is considered the main brain area to control eye movements and visual attention in normal binocular humans (Silvanto et al., 2006; Thiebaut de Schotten et al., 2011). There is no correlation between the poor cVA of amblyopic eye and V1 structure and function in SA patients. The function of right IPS in AA also showed a positive correlation with the reduced LogMAR cVA of anisometropic amblyopic eye, probably because the less perturbed eye movements function of AA patients did not correlate with FEF disfunction, but the unclear visual signal resulted in abnormal visual transmission in parietal lobe (Felleman and Van Essen, 1991; Friston and Buchel, 2000), and caused the correlation between function of IPS and cVA of amblyopic eye in AA patients.

The major limitations of this study were the fact that the laterality of the amblyopic eyes was not controlled in this study, with 13 Left amblyopic eye and 7 right dominant eyes in both SA and AA groups. This predominance of LE amblyopes with perturbed ocular motility and ability to drive attention would also be expected to result in less activation of the left ipsilateral visual attention network and enhanced dominant right eyes (13/20 for each group) activation of the right hemisphere visual attention network. This laterality bias would also have further confounded the right hemisphere dominance of visual attention. Hence the second limitation of this study was our focus on the key visual attention-related brain areas, rather than function and FC in other brain areas such as visual areas *per se*. Our choice of ROI was based on prior research relating to the vast oculomotor literature and the association between impaired eye movements, saccadic reaction times and visual attention-related functions in strabismic and anisometropic amblyopes.

Regarding laterality statistical measures were utilized to minimize its effects. Comparison of left SA patients and right SA patients indicated that there was no statistical difference in activation value as measured with zALFF and zReHo in all the six ROIs. The comparison results of Left AA and right AA patients are similar, and the comparison results of left non-dominant eye HC and right non-dominant eye HC area also similar (Supplementary Table 1) and so, the left and right amblyopic eye patients were considered as one group and left and right non-dominated eye HC also. Furthermore the left or right amblyopic eyes were coded as 1 or 2 and made concomitant variables in the statistical process to reduce the effect of the problem while bearing in mind that each eye projects to both hemispheres (Leguire et al., 1990; Zhang et al., 2005).

Future studies of resting state FC would benefit greatly from a more rigorous considerations of the importance of ocular laterality and a more in-depth testing of temporal ocular motility for each participant prior to comparison of GMV and activation densities in strabismic and anisometropic amblyopia. Lastly inclusion of other ROIs along the visual attention networks in particular frontal cortical areas, as sites of top-down visually driven cognition and middle temporal (MT) which is involved in activation of bottom-up visual attention and eye movements.

## Conclusion

This study has demonstrated that resting-state visual attention network function was impaired in SA and AA patients though probably more so in the SA participants with worse clinical VA and likely worse ocular motility and least activation of both V1 areas. The right FEF and right IPS areas of both the SA and AA groups showed higher activation than those of the HC group but lower activation than those of the HC group in the left FEF area. Both the V1–right FEF and right IPS–right FEF functional pathways showed higher connectivity in the SA and AA groups than in the HC group suggesting that the 13/20 right eye dominant participants also usually showed enhanced monocular activation of their ipsilateral hemisphere attention networks. GM increased in both V1 areas in the SA group compared with the HC group suggesting less refinement of binocular synapses and connections across the corpus callosum.

## Data Availability Statement

The raw data supporting the conclusions of this article will be made available by the authors, without undue reservation.

## Ethics Statement

The studies involving human participants were reviewed and approved by the Human Ethics Committees of La Trobe University, Melbourne, VIC, Australia and Southwest Hospital, Chongqing, China. The patients/participants provided their written informed consent to participate in this study.

## Author Contributions

HW: data analysis and manuscript writing. ML and JW: data collection. SC: research guiding and manuscript writing. ZY and TY: data analysis and research guiding. DC: research guiding. All authors contributed to the article and approved the submitted version.

## Conflict of Interest

The authors declare that the research was conducted in the absence of any commercial or financial relationships that could be construed as a potential conflict of interest.

## Publisher’s Note

All claims expressed in this article are solely those of the authors and do not necessarily represent those of their affiliated organizations, or those of the publisher, the editors and the reviewers. Any product that may be evaluated in this article, or claim that may be made by its manufacturer, is not guaranteed or endorsed by the publisher.

## Footnotes

1
http://dbm.neuro.uni-jena.de/vbm8/

2
http://www.restfmri.net/forum/DPARSF

3
http://www.fil.ion.ucl.ac.uk/spm

4
http://restfmri.net/forum/RESTplusV1.2

5
https://www.nitrc.org/projects/bnv
