# Supplementary material for: Functional Deficits and Structural Changes Associated With the Visual Attention Network During Resting State in Adult Strabismic and Anisometropic Amblyopes
Source: Front Hum Neurosci. 2022 May 18;16:862703. doi: 10.3389/fnhum.2022.862703 (PMC9157425; doi:10.3389/fnhum.2022.862703)
Supplement: Supplementary file 1 [file Data_Sheet_1.PDF]

Supplementary table 1. The comparison *P* value of left and right amblyopic/non-dominate eye of SA/AA/HC group in the six ROIs by ANOVA

	SA zALFF	AA zALFF	HC zALFF	SA zReHo	AA ReHo	HC zReHo
Left FEF	0.298	0.765	0.270	0.438	0.975	0.880
Right FEF	0.904	0.992	0.332	0.454	0.851	0.271
Left IPS	0.542	0.227	0.742	0.384	0.551	0.348
Right IPS	0.993	0.893	0.479	0.090	0.870	0.470
Left V1	0.235	0.595	0.130	0.632	0.291	0.636
Right V1	0.964	0.266	0.219	0.782	0.331	0.316

There is no statistic difference in all ROIs of the left and right amblyopic/dominate eye of SA/AA/HC group in the six ROIs. Therefore, the left/right amblyopic/non-dominate eye patients/controls could be considered as one group.
